# The Impact of Low-Volume High-Intensity Interval Training (LV-HIIT) on Fatty Liver Index (FLI) and Estimated Glomerular Filtration Rate (eGFR) in Patients with Type 2 Diabetes Mellitus (T2DM)

**DOI:** 10.2174/0115733998320832240805113238

**Published:** 2024-08-12

**Authors:** Rasoul Raesi, Saeid Kalbasi, Abbas Ali Gaeini, Maryam Haji Ghasem Kashani, Khadijeh Tajik

**Affiliations:** 1 Department of Nursing, Torbat Jam Faculty of Medical Sciences, Torbat Jam, Iran;; 2 Department of Health Services, School of Health, Management, Mashhad University of Medical Sciences, Mashhad, Iran;; 3 Department of Endocrinology, Loghman Hospital, Shahid Beheshti University of Medical Sciences, Tehran, Iran;; 4 Department of Exercise Physiology, Faculty of Physical Education and Exercise Sciences, University of Tehran, Tehran, Iran;; 5 Department of Cellular and Molecular Biology, School of Biology and Institute of Biological Sciences, Damghan University, Damghan, Iran;; 6 Department of Physical Education and Sport Sciences, Alborz Pardis of Tehran University, Tehran, Iran

**Keywords:** Diabetes, fatty liver Index (FLI), high-intensity interval training (HIIT), exercise, estimated glomerular filtration Rate (eGFR), type 2 diabetes (T2DM), kidney, glomerulus, glycosylated hemoglobin (HbA1C), fasting blood sugar (FBS)

## Abstract

**Background:**

Prevention and reduction of liver fat accumulation and maintenance of Glomerular Filtration Rate (GFR) have been proposed as important therapeutic goals in patients with Type 2 Diabetes Mellitus (T2DM).

**Aim:**

This study aimed to determine the effect of Low-Volume High-Intensity Interval Training (LV-HIIT) on fatty liver index (FLI) and GFR estimation in patients with T2DM.

**Methods:**

This randomized controlled trial included 80 patients with T2DM and a sedentary lifestyle, randomly divided into HIIT (n=40) and a control group (n=40). Patients with a history of T2DM for at least one year and HbA1C levels between 6.4% and 10% were selected. The intervention group underwent a 4-week LV-HIIT course, comprising 3 sessions per week, while the control group did not receive any intervention. FLI, eGFR, anthropometric measurements, and laboratory variables were assessed in all participants before and after the intervention.

**Results:**

FLI (62.0 at baseline, 53.0 at follow-up) significantly decreased in the LV-HIIT group after the intervention, while eGFR (71.0 at baseline, 73.6 at follow-up) significantly increased (*P<*0.001). However, the control group showed a significant reduction only in Fasting Blood Sugar (FBS) (*P<*0.05). After the intervention, the LV-HIIT group had significantly lower FBS (129.0 at baseline, 121.0 at follow-up), Alanine Aminotransferase (ALT) (24.0 at baseline, 18.0 at follow-up), and Gamma-Glutamyl Transferase (GGT) (22.0 at baseline, 19.0 at follow-up), as well as higher eGFR, compared to the control group (*P<*0.05).

**Conclusions:**

LV-HIIT exercise appears to be a promising and effective training method for improving FLI and eGFR in patients diagnosed with T2DM.

**Clinical Trial Registration:**

IRCT 20200 729048246N1.

## 1 INTRODUCTION

Type 2 diabetes mellitus, often referred to as T2DM, is a chronic metabolic disorder characterized by high levels of blood sugar resulting from insulin resistance and relative insulin deficiency [1]. This type of diabetes accounts for the majority of diabetes cases worldwide, affecting approximately 90-95% of individuals with diabetes [2]. Type 2 diabetes mellitus (T2DM) commonly occurs in adults, but there is a growing trend of diagnosis in children and adolescents. This is largely attributed to the increasing prevalence of obesity and sedentary behaviors among younger populations [3]. The exact cause of T2DM is multi-factorial, involving a complex interplay of genetic predisposition, environmental factors, and lifestyle choices [4].

In individuals with T2DM, cells become resistant to the action of insulin, leading to elevated blood sugar levels. Over time, the pancreas is unable to produce enough insulin to compensate for this resistance, resulting in further increases in blood sugar levels [4, 5]. T2DM is a multifaceted condition that can lead to numerous complications, such as cardiovascular disease, neuropathy, nephropathy, and retinopathy if left unmanaged [6, 7]. The long-term consequences of T2DM pose a significant burden on individuals, healthcare systems, and economies worldwide [8].

Effective management strategies, including lifestyle modifications, pharmacological interventions, and regular monitoring, are crucial in mitigating the impact of T2DM and its associated complications [9, 10]. The incidence of Type 2 Diabetes Mellitus (T2DM) has been on the rise, primarily attributed to sedentary lifestyles, poor dietary choices, and obesity. These factors have collectively contributed to the escalating rates of T2DM in recent years [11]. T2DM is characterized by the body's inability to effectively utilize in two significant complications associated with T2DM are fatty liver disease and impaired renal function, both of which can have severe consequences on overall health and quality of life [12-14].

Fatty liver disease, also known as non-alcoholic fatty liver disease (NAFLD), is a condition characterized by the accumulation of excess fat in the liver cells [15]. NAFLD is closely linked to obesity, insulin resistance, and T2DM, and its prevalence is higher in individuals with T2DM compared to the general population [16, 17]. NAFLD can progress to more severe forms, such as non-alcoholic steatohepatitis (NASH), which can lead to liver fibrosis, cirrhosis, and potentially liver failure [18]. Another complication related to diabetes is kidney damage which disrupts the amount of glomerular filtration [19, 20]. The glomerular filtration rate (GFR) is a key indicator of kidney function, reflecting the rate at which blood is filtered by the glomeruli in the kidneys [21]. In T2DM, prolonged exposure to high blood sugar levels can damage the kidneys' filtering units, leading to a decline in GFR and the development of diabetic nephropathy. Impaired kidney function can result in serious complications, including end-stage renal disease, necessitating dialysis or kidney transplantation [22, 23].

Furthermore, to mitigate the impact of these complications, lifestyle modifications, including regular physical activity and exercise, have been recognized as essential components of T2DM management [24-26]. One particular exercise modality that has gained significant attention in recent years is low-volume high-intensity interval training (LV-HIIT). LV-HIIT is a time-efficient form of exercise that involves short bursts of high-intensity exercise alternated with periods of low-intensity recovery or rest [27, 28]. Despite the low overall exercise volume, LV-HIIT has been shown to elicit numerous health benefits, including improved glycemic control, increased insulin sensitivity, and favorable changes in body composition [29, 30]. Several studies have investigated the potential effects of LV-HIIT on fatty liver disease and renal function in individuals with T2DM. The study's results by Mousa Khalafi and colleagues (2021) suggest that HIIT could improve the liver fat of overweight and obese adults with metabolic disorders despite no weight loss [31]. Toyama *et al.* showed that exercise therapy could modify lipid metabolism and improve the estimated glomerular filtration rate (eGFR) in patients with cardiovascular disease and CKD [32]. Furthermore, one meta-analysis study showed that exercise therapy could increase eGFR by 2.62 ml/min/1.73m^2^ in non-dialysis CKD patients [33]. Moinuddin and Leehey recently showed that aerobic exercise increases the estimated glomerular filtration rate (eGFR) in CKD [34].

A previous study has suggested that HIIT is superior to moderate-intensity exercising in reducing the risk of NAFLD, liver fibrosis, and liver enzyme elevation [35]. The mechanisms underlying the beneficial effects of LV-HIIT on fatty liver disease and renal function in T2DM are not fully understood but are likely multifactorial. Proposed mechanisms include improved insulin sensitivity, reduced visceral adiposity, decreased inflammation, and enhanced endothelial function [36, 37].

Therefore, careful monitoring of renal and liver function is of great clinical importance in patients with T2DM disease. These patients should undergo accurate evaluations of the FLI and eGFR, which are the available indicators of NAFLD and CKD. Consequently, Prevention and reduction of liver fat accumulation and maintenance of GFR have been proposed as important therapeutic goals in patients with T2DM.

Previous studies have highlighted the benefits of HIIT in improving cardiovascular fitness, insulin sensitivity, and overall metabolic health in individuals with T2DM. However, the specific effects of LV-HIIT on the Fatty Liver Index and eGFR in this population remain relatively unexplored. The primary aim of this study is to investigate the impact of LV-HIIT on the Fatty Liver Index and eGFR in patients with T2DM. Moreover, by assessing changes in these parameters following a structured LV-HIIT intervention, this research aims to elucidate the potential benefits of this exercise modality in improving liver and kidney health in individuals with T2DM. Understanding the effects of LV-HIIT on these specific markers could provide valuable insights into the role of exercise in the management of T2DM-related complications beyond glycemic control.

The investigation into the impact of LV-HIIT on the Fatty Liver Index and eGFR in patients with T2DM holds significant promise in advancing our understanding of exercise-based interventions for metabolic disorders. In addition, by focusing on these key parameters, this study aims to contribute valuable data to the existing body of research on the benefits of LV-HIIT in improving liver and kidney health in individuals with T2DM. The findings of this research have the potential to inform clinical practice and enhance the management strategies for patients with T2DM, ultimately improving their overall health outcomes. Therefore, the current study aimed to investigate the effect of Low-Volume High-Intensity Interval Training (LV-HIIT) on the Fatty Liver Index (FLI) and estimated GFR in patients with T2DM.

## 2 METHODS AND MATERIALS

This experimental study was conducted on 80 patients with type 2 diabetes in the age range of 40-65 years who were referred to the Diabetes and Metabolic Diseases Clinic of Loghman Hakim Hospital, Tehran, and were willing to participate in the study voluntarily.

The inclusion criteria were patients with a history of T2DM for at least one year and a glycosylated hemoglobin (HbA1C) level of 6.4%-10% and who did not exercise regularly in the previous 6 months. Moreover, the exclusion criteria included patients with a history of functional limitations (such as osteoarthritis), respiratory, inflammatory, cardiovascular, renal, and other chronic diseases, smoking, or heavy drinking. After explaining the subject, objectives, and research methods, completing and obtaining the consent form, and completing the health and sports history questionnaire and examination by a physician, the subjects were randomly divided into two groups of HIIT (n=40) and control (n=40) after fulfilling the mentioned conditions. It is noteworthy that the number and volume of samples based on research backgrounds were used in interventions related to sports activities.

### 2.1 Exercise Intervention

The intervention group underwent a 4-week course of LV-HIIT, which included 3 sessions per week. The exercise was performed using a treadmill (1790 CLUB HEALTH STREAM).

Each session included 3 parts: warm-up, main part, and cool-down. Moreover, the main part included four 1-minute intervals of exercising with an intensity of 70% HR_max_ that were separated by three 4-minute intervals of exercising with an intensity of 60% HR_max_. Then, the session ended with a 5-minute cool-down.

#### 2.1.1 Blood Sample Analysis

Blood samples were collected from the vein in the forearm in two stages, one day before the first training session (pre-test) and 24 hours after the last training session, and after 10 -12 hours of fasting.

The lipid profile, including Low-Density Lipoprotein (LDL), total cholesterol, High-Density Lipoprotein (HDL), and triglyceride, was assessed using colorimetric enzyme reactions and an automatic chemical analyzer (Cobas C111; Roche Diagnostics, Indianapolis, IN, USA). Moreover, the HbA_1c_ levels of the patients were assessed using the anion exchange chromatography, while the plasma glucose levels were assessed using a specific Enzyme-Linked Immunosorbent Assay (ELISA) kit (Mercodia, Sylveniusgatan 8A, Uppsala, Sweden). Also, ALT, AST, and GGT levels were assessed using the DELTA DARMAN PART kits (Pars Azmon, Tehran, Iran) based on the manufacturer's instructions. Finally, serum creatinine (Cr) levels were assessed using an enzymatic kit (Creatinine plus Kit, RocheLife Science, US), while the Blood Urea Nitrogen (BUN) was assessed using the urease test (Urease UV rate method, RocheLife Science, US). The plasma values were measured by the ELISA method using kits made by Elabscience (USA) (Fetuin -B kit with sensitivity and range of 56.25 pg/mL and 93.75 -6000 pg/mL) and (RBP4 kit with sensitivity and range of 0.94 ng/mL and 1.56 -100 ng/mL). Moreover, insulin was evaluated using the ELISA method by kits made by Elabscience (USA) with sensitivity and range of 0.47 µIU/ml and 0.78 -50 µIU/ml.

#### 2.1.2 Fatty Liver Index

As a non-invasive method for assessing hepatic steatosis, the FLI of each participant was calculated using the relevant formula [38]:

##### 2.1.2.1 Glomerular Filtration Rate

The estimated GFR of each patient was calculated using the Modification of Diet in Renal Disease (MDRD) formula recommended by the American Diabetes Association:


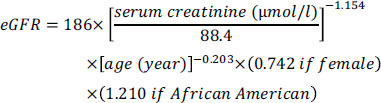


#### 2.1.3 Statistical Analysis

Statistical analysis was performed using the SPSS statistical software (version 18.0; SPSS Inc., Chicago, IL, USA). The continuous variables with normal distribution were described using the mean and Standard Deviation (SD), while the median, first quartile, and third quartile were used for those without normal distribution. Moreover, intergroup comparisons were performed using the independent samples t-test and Mann-Whitney test for normally and non-normally distributed variables, respectively. Likewise, variables with normal distribution underwent intragroup comparisons using the paired samples t-test, while those without normal distribution were compared using the Wilcoxon test. Finally, the Mann-Whitney test was used for intergroup comparisons of the progress rates of study variables. The significance level was considered at 0.05 for all comparisons.

## 3 RESULTS

In our study, we initially observed no statistically significant differences in any of the study variables between the groups prior to the intervention (*P>*0.05), except for creatinine levels, which were notably lower in the control group compared to the LV-HIIT group (*P<*0.05). However, following the intervention, substantial changes were evident.

Firstly, the LV-HIIT group exhibited a significant reduction in Fatty Liver Index (FLI) when compared to their baseline values (62.0 at baseline, 53.0 at follow-up, *P<*0.001). Furthermore, the estimated Glomerular Filtration Rate (eGFR) displayed a significant increase solely in the LV-HIIT group after the intervention (71.0 at baseline, 73.6 at follow-up, *P<*0.001), with no such change observed in the control group (*P>*0.05) (Tables **1-3**). Moreover, the LV-HIIT group exhibited substantial post-intervention decreases in various anthropometric measurements. These included waist-to-hip ratio (Table **3**, *P<*0.001), waist circumference (Table **3**, *P<*0.001), Body Mass Index (BMI) (Table **3**, *P<*0.001), and overall body weight (Table **3**, *P<*0.001). In contrast, the control group showed no such decreases (*P>*0.05). Turning to the lipid profile, the LV-HIIT group demonstrated significant decreases in total cholesterol (Table **3**, *P<*0.001), triglyceride levels (Table **3**, *P<*0.001), and LDL cholesterol (Table **3**, *P*=0.001) after the intervention, in contrast to the control group (*P>*0.05). Finally, BUN (Table **3**, *P<*0.001) and creatinine (Table **3**, *P<*0.001) levels notably decreased in the LV-HIIT group after the intervention, whereas this pattern did not emerge in the control group (*P>*0.05).

## 4 DISCUSSION

The current study aimed to investigate the effect of Low-Volume High-Intensity Interval Training (LV-HIIT) on the Fatty Liver Index (FLI) and estimated GFR in patients with T2DM. The primary outcome of this study demonstrates that four weeks of LV-HIIT yielded improvements in the FLI and eGFR, which were associated with modifications in lipid metabolism among individuals with T2DM. Specifically, FLI significantly decreased after the four-week LV-HIIT intervention. These findings align with a study by Ray et al., which revealed that daily aerobic exercise lasting 50 to 60 minutes over a four-week period led to enhanced insulin sensitivity, increased glucose oxidation, and reduced visceral fat, with exercise intensity progressing from 60%–65% of maximum heart rate to 80%–85% of maximum heart rate [39]. Furthermore, Nathan *et al.* reported that obese individuals with non-alcoholic fatty liver disease (NAFLD) experienced a significant reduction in hepatic steatosis after four weeks of High-Intensity Interval Training (HIIT) involving 4-minute intervals at 80% of VO2max followed by 3-minute active recovery at 50% of VO2max [40]. These findings suggest that the specific mode of exercise plays a crucial role in the impact of HIIT on liver function tests (LFT). Additionally, the effect of exercise on FLI is likely mediated through mechanisms such as weight loss, reduced insulin resistance, and improvements in HbA1c and atherogenic dyslipidemia associated with metabolic syndrome [41-43]. HIIT may also ameliorate NAFLD by inhibiting inflammatory pathways like NF-κB and AMPK [44, 45].

Studies have previously demonstrated that individuals with nonalcoholic steatohepatitis exhibit decreased eGFR [46], and patients with chronic kidney disease (CKD) often present lower levels of HDL and higher triglycerides compared to healthy counterparts [32]. Additionally, fatty liver may harm renal function by means of oxidized LDLs, which, when combined with proteins, lead to glomerular injury and mesangial cell proliferation [46]. Both HIIT and moderate-intensity continuous aerobic exercise have shown the potential to improve renal function. Therefore, it is reasonable to propose that appropriate exercise regimens can shield the kidneys from diabetes-induced renal complications [47].

Furthermore, this study observed significant reductions in Fasting Blood Sugar (FBS) and Hemoglobin A1c (HbA1c) levels following the four-week HIIT course. Previous research supports this, as a study involving 20-minute submaximal heart rate cycling or running exercises, coupled with 20-minute warm-up/cool-down sessions three times a week for four weeks, revealed reduced insulin resistance and blood glucose levels in individuals with type 2 diabetes [39]. The beneficial impact of exercise on glycemic control in individuals with type 2 diabetes may be attributed to its ability to increase glucose uptake mediated by insulin and the quantity of glucose transporters (GLUT4) through specific signaling pathways [48]. Other mechanisms involved in HIIT can be the effect of exercise on the pathway of antioxidant enzymes [49]. This study indicates that a four-week regimen of LV-HIIT can significantly improve various health parameters, including waist-to-hip ratio, waist circumference, Body Mass Index (BMI), weight, FBS, HbA1c, ALT, GGT, LDL, triglycerides, total cholesterol, Blood Urea Nitrogen (BUN), creatinine, FLI, and eGFR among patients with type 2 diabetes. Consequently, this non-pharmacological intervention holds promise as an effective modality for mitigating diabetes-related complications and enhancing renal and liver function.

## CONCLUSION AND RECOMMENDATIONS

The findings of the present study highlight the significant impact of a 4-week course of Low-Volume High-Intensity Interval Training (LV-HIIT) on various metabolic and physiological parameters in patients with Type 2 Diabetes Mellitus (T2DM). The observed improvements in waist-to-hip ratio, waist circumference, BMI, weight, fasting blood sugar (FBS), HbA1c, liver enzymes (ALT, GGT), lipid profile (LDL, triglycerides, total cholesterol), renal function markers (BUN, creatinine), Fatty Liver Index (FLI), and estimated Glomerular Filtration Rate (eGFR) underscore the comprehensive benefits of LV-HIIT as a non-pharmacological intervention for managing diabetes-related complications. Furthermore, the results suggest that LV-HIIT can serve as a valuable method for enhancing both liver and kidney function in individuals with T2DM. In addition, by targeting multiple aspects of metabolic health, including glucose control, lipid metabolism, liver fat content, and renal function, LV-HIIT demonstrates its potential as a holistic approach to improving the overall well-being of diabetic patients. The ability of LV-HIIT to positively impact FLI and eGFR underscores its efficacy in addressing key components of T2DM management beyond traditional pharmacological interventions.

The promising outcomes of LV-HIIT exercise in improving FLI and eGFR in T2DM patients signify a shift towards personalized and effective exercise prescriptions in diabetes care. The multifaceted benefits observed in this study support the integration of LV-HIIT into the treatment paradigm for individuals with T2DM, offering a tailored approach to enhancing metabolic health and reducing the burden of diabetes-related complications. These findings emphasize the importance of incorporating structured exercise regimens like LV-HIIT as a cornerstone of comprehensive diabetes management strategies aimed at optimizing patient outcomes and quality of life.

## LIMITATIONS OF THE STUDY

The results of this research are related to patients with type 2 diabetes referred to the Diabetes and Metabolic Diseases Clinic of Loghman Hakim Hospital in Tehran, Iran, therefore the generalization of the findings should be performed with caution. Furthermore, the lack of information related to a marker of kidney disease such as albuminuria in this research is a significant bias that sets a special limitation in the findings. Additionally, the effect of physical activity on muscle metabolism and creatinine production is one of the other things that have not been investigated in this research. Therefore, other researchers are recommended to consider these things in their future studies. Among the other limitations of the present study's limitations were the short study period and the small number of samples. It is suggested that future studies be investigated over a longer time and with a larger sample size. The limitations of the research create caution in the field of generalization of the findings, therefore, caution should be observed in the generalization of the findings.

## Figures and Tables

**Table 1:** Demographic information of patients with type 2 diabetes.

**Variable**	**Group**	**Intervention**	**Control**	**Total**	***P*-value**
Age (years)	-	51.55±5.93	54.40±7.88	-	0.071
Gender n(%)	male	15(37.5)	19(47.5)	34(42.5)	0.366
	female	25(62.5)	21(52.5)	46(57.5)	-
-	Total	40(100.0)	40(100.0)	80(100.0)	-

**Note: ** Descriptive statistics are reported by mean ± SD.

**Table 2:** Intergroup comparisons of study variables.

**Variable**	**Assessment**	**LV-HIIT group**	**Control Group**	***P*-value**
Weight (kg) ^β^	Pre-intervention	68.0 (62.5, 79.6)	73.1 (61.2, 76.8)	0.865 ^δ^
	Post-intervention	66.0 (61.2, 75.8)	73.2 (61.0, 77.0)	0.281 ^δ^
BMI (kg/m^2^) ^β^	Pre-intervention	26.9 (25.1, 29.4)	25.2 (24.1, 31.9)	0.533 ^δ^
	Post-intervention	26.2 (24.4, 27.5)	25.3 (24.1, 30.8)	0.887 ^δ^
Waist circumference (cm) ^β^	Pre-intervention	96.0 (61.5, 107.5)	98.5 (93.2, 100.0)	0.359 ^δ^
	Post-intervention	94.0 (90.5, 100.0)	98.5 (93.2, 101.0)	0.024 ^δ^*
Waist-to-hip ratio ^β^	Pre-intervention	0.94 (0.90, 0.97)	0.93 (0.90, 0.97)	0.977 ^δ^
	Post-intervention	0.93 (0.89, 0.96)	0.93 (0.91, 0.97)	0.315 ^δ^
FBS (mg/dL) ^β^	Pre-intervention	129.0 (126.5, 171.5)	131.0 (116.5, 162.2)	0.302 ^δ^
	Post-intervention	121.0 (104.0, 145.0)	130.0 (120.5, 171.5)	0.014 ^δ^*
HbA_1_c (%) ^β^	Pre-intervention	7.0 (6.7, 7.7)	6.7 (6.1, 7.5)	0.073 ^δ^
	Post-intervention	6.5 (6.2, 6.9)	6.6 (6.1, 7.5)	0.320 ^δ^
LDL (mg/dL) ^α^	Pre-intervention	95.75 ± 33.2	84.22 ± 25.3	0.084 ^γ^
	Post-intervention	83.99 ± 27.5	88.42 ± 29.1	0.484 ^γ^
HDL (mg/dL) ^β^	Pre-intervention	40.0 (35.5, 43.5)	42.5 (34.0, 53.2)	0.226 ^δ^
	Post-intervention	39.9 (38.0, 44.5)	44.5 (37.0, 54.2)	0.307 ^δ^
Triglyceride (mg/dL) ^β^	Pre-intervention	124.0 (110.0, 229.0)	122.0 (105.0, 214.7)	0.326 ^δ^
	Post-intervention	112.0 (80.0, 195.5)	124.0 (94.0, 213.7)	0.209 ^δ^
Total cholesterol (mg/dL) ^α^	Pre-intervention	175.0 ± 27.8	171.8 ± 33.5	0.639 ^γ^
	Post-intervention	156.2 ± 36.9	169.9 ± 34.4	0.090 ^γ^
AST (IU/L) ^β^	Pre-intervention	23.0 (18.0, 30.5)	20.0 (17.2, 30.0)	0.650 ^δ^
	Post-intervention	22.0 (17.5, 28.0)	21.5 (16.2, 32.2)	0.538 ^δ^
ALT (IU/L) ^β^	Pre-intervention	24.0 (15.0, 34.0)	21.0 (13.7, 33.0)	0.865 ^δ^
	Post-intervention	18.0 (14.0, 25.0)	22.5 (15.5, 38.5)	0.041 ^δ^*
GGT (IU/L) ^β^	Pre-intervention	22.0 (15.5, 31.5)	20.0 (15.2, 30.7)	0.514 ^δ^
	Post-intervention	19.0 (12.5, 23.0)	21.0 (19.0, 28.7)	0.025 ^δ^*
BUN (mg/dL) ^α^	Pre-intervention	29.22 ± 6.57	27.13 ± 8.48	0.218 ^γ^
	Post-intervention	24.60 ± 5.91	26.31 ± 9.19	0.327 ^γ^
Creatinine (mg/dL) ^β^	Pre-intervention	1.0 (1.0, 1.1)	0.9 (0.8, 1.1)	0.017 ^δ^*
	Post-intervention	1.0 (0.9, 1.1)	0.9 (0.8, 1.13)	0.795 ^δ^
FLI ^β^	Pre-intervention	62.0 (30.0, 82.0)	51.0 (18.0, 66.0)	0.205 ^δ^
	Post-intervention	53.0 (22.5, 72.0)	52.5 (19.7, 71.7)	0.623 ^δ^
GFR (mL/min/1.73m^2^) ^β^	Pre-intervention	71.0 (61.4, 76.7)	71.6 (64.9, 77.0)	0.643 ^δ^
	Post-intervention	73.6 (69.1,76.1)	69.7 (62.6, 74.9)	0.021 ^δ^*

**Note:** ^α^Data are presented as mean ± SD. ^β^ Data are presented as median (quartile 1, quartile 3). ^γ^* P*-value calculated using the paired samples t-test. ^δ^* P*-value calculated using the Mann-Whitney test. * Significant difference (*P<*0.05).

**Table 3:** Within-group comparisons of study variables.

**Variable**	**Group**	**Pre-intervention**	Post-Intervention	***P*-value**
Weight (kg) ^β^	LV-HIIT	68.0 (62.5, 79.6)	66.0 (61.2, 75.8)	<0.001 ^δ^*
	Control	73.1 (61.2, 76.8)	73.2 (61.0, 77.0)	0.102 ^δ^
BMI (kg/m^2^) ^β^	LV-HIIT	26.9 (25.1, 29.4)	26.2 (24.4, 27.5)	<0.001 ^δ^*
	Control	25.2 (24.1, 31.9)	25.3 (24.1, 30.8)	0.233 ^δ^
Waist circumference (cm) ^β^	LV-HIIT	96.0 (61.5, 107.5)	94.0 (90.5, 100.0)	<0.001 ^δ^*
	Control	98.5 (93.2, 100.0)	98.5 (93.2, 101.0)	0.085 ^δ^
FBS (mg/dL)^β^	LV-HIIT	129.0 (126.5, 171.5)	121.0 (104.0, 145.0)	<0.001 ^δ^*
	Control	131.0 (116.5, 162.2)	130.0 (120.5, 171.5)	0.002 ^δ^*
HbA_1_c (%)^β^	LV-HIIT	7.0 (6.7, 7.7)	6.5 (6.2, 6.9)	<0.001 ^δ^*
	Control	6.7 (6.1, 7.5)	6.6 (6.1, 7.5)	0.306 ^δ^
LDL (mg/dL)^α^	LV-HIIT	95.75 ± 33.2	83.99 ± 27.5	0.001 ^γ^*
	Control	84.22 ± 25.3	88.42 ± 29.1	0.007 ^γ^*
HDL (mg/dL)^β^	LV-HIIT	40.0 (35.5, 43.5)	39.9 (38.0, 44.5)	0.075 ^δ^
	Control	42.5 (34.0, 53.2)	44.5 (37.0, 54.2)	0.144 ^δ^
Triglyceride (mg/dL)^β^	LV-HIIT	124.0 (110.0, 229.0)	112.0 (80.0, 195.5)	<0.001 ^δ^*
	Control	122.0 (105.0, 2147)	124.0 (94.0, 213.7)	0.079 ^δ^
Total cholesterol (mg/dL)^α^	LV-HIIT	175.0 ± 27.8	156.2 ± 36.9	<0.001 ^γ^*
	Control	171.8 ± 33.5	169.9 ± 34.4	0.657 ^γ^
AST (IU/L)^β^	LV-HIIT	23.0 (18.0, 30.5)	22.0 (17.5, 28.0)	0.209 ^δ^
	Control group	20.0 (17.2, 30.0)	21.5 (16.2, 32.2)	0.184 ^δ^
ALT (IU/L)^β^	LV-HIIT group	24.0 (15.0, 34.0)	18.0 (14.0, 25.0)	<0.001 ^δ^*
	Control group	21.0 (13.7, 33.0)	22.5 (15.5, 38.5)	0.056 ^δ^
1GGT (IU/L)^β^	LV-HIIT group	22.0 (15.5, 31.5)	19.0 (12.5, 23.0)	0.012 ^δ^*
	Control group	20.0 (15.2, 30.7)	21.0 (19.0, 28.7)	0.777 ^δ^
BUN (mg/dL)^α^	LV-HIIT group	29.22 ± 6.57	24.60 ± 5.91	<0.001 ^γ^*
	Control group	27.13 ± 8.48	26.31 ± 9.19	0.131^γ^
Creatinine (mg/dL)^β^	LV-HIIT group	1.0 (1.0, 1.1)	1.0 (0.9, 1.1)	<0.001 ^δ^*
	Control group	0.9 (0.8, 1.1)	0.9 (0.8, 1.13)	0.098^δ^
FLI^β^	LV-HIIT group	62.0 (30.0, 82.0)	53.0 (22.5, 72.0)	<0.001 ^δ^*
	Control group	51.0 (18.0, 66.0)	52.5 (19.7, 71.7)	0.001^δ^*
GFR (mL/min/1.73m^2^) ^β^	LV-HIIT group	71.0 (61.4, 76.7)	73.6 (69.1,76.1)	<0.001 ^δ^*
	Control group	71.6 (64.9, 77.0)	69.7 (62.6, 74.9)	0.133 ^δ^

**Note:** ^α^Data are presented as mean ± SD. ^β^ Data are presented as median (quartile 1, quartile 3). ^γ^* P*-value calculated using the paired samples t-test. ^δ^* P-*value calculated using the Wilcoxon test. * Significant difference (*P<*0.05).

## Data Availability

The data that support the findings of this study are available from the corresponding author upon reasonable request.

## LIST OF ABBREVIATIONS

ALT: Alanine Aminotransferase
FLI: Fatty Liver Index
GFR: Glomerular Filtration Rate
GGT: Gamma-Glutamyl Transferase
T2DM: Type 2 Diabetes Mellitus

## STANDARDS OF REPORTING

CONSORT guidelines were followed.
